# Signatures and Clinical Significance of Amino Acid Flux in Sarcopenia: A Systematic Review and Meta-Analysis

**DOI:** 10.3389/fendo.2021.725518

**Published:** 2021-09-13

**Authors:** Miao Dai, Taiping Lin, Jirong Yue, Lunzhi Dai

**Affiliations:** ^1^Department of Geriatrics and National Clinical Research Center for Geriatrics, West China Hospital of Sichuan University, Chengdu, China; ^2^Department of State Key Laboratory of Biotherapy, West China Hospital of Sichuan University, and Collaborative Innovation Center of Biotherapy, Chengdu, China

**Keywords:** sarcopenia, metabolomics, amino acid, biomarkers, meta-analysis

## Abstract

**Background:**

Dysregulation of amino acids is closely linked to the initiation and progression of sarcopenia. We summarized recent advancements in the studies of amino acid profiles in sarcopenia and systematically presented the clinical significance of amino acid flux in sarcopenia.

**Methods:**

We systematically searched in MEDLINE, EMBASE, and Cochrane library from inception to June 1, 2021 to capture all studies examining metabolomics of sarcopenia. We used the following keywords: sarcopenia, metabonomics, metabolomics, amino acid profile, and mass spectrometry. Original articles comparing amino acid patterns between persons with and without sarcopenia were included. Two independent investigators independently completed title and abstract screening, data extraction, and quality evaluation. We used a random effects model to examine the association between amino acids levels and sarcopenia. Sensitivity analyses restricted the analyses to studies in which muscle mass was measured by bioelectrical impedance analysis. Study quality was evaluated according to the Agency for Healthcare Research and Quality (AHRQ) checklist.

**Results:**

The systematic research yielded six eligible articles, comprising 1,120 participants. Five studies used muscle mass in combination with physical performance and/or muscle strength as the criteria to diagnose sarcopenia, while one study used muscle mass as a diagnostic criterion alone. We found that the concentrations of branched-chain amino acids leucine (standardized mean difference [SMD] -1.249; 95% confidence interval [CI]: -2.275, -0.223, *P* = 0.02, I^2^ = 97.7%), isoleucine (SMD -1.077; 95% CI: -2.106, -0.049, *P* = 0.04, I^2^ = 97.8%), and aromatic amino acid tryptophan (SMD -0.923; 95% CI: -1.580, -0.265, *P* = 0.01, I^2^ = 89.9%) were significantly reduced in individuals with sarcopenia. Study results were robust in sensitivity analysis.

**Conclusions:**

The homeostasis of amino acids is critical to maintaining muscle health. The profiles of amino acids might be useful biomarkers for the characterization of sarcopenia. Future studies are warranted to study the clinical significance of amino acids in the diagnosis and treatment of sarcopenia.

## Introduction

Sarcopenia, defined as the age-related progressive loss of skeletal muscle strength, mass, and/or function (1), has been established as a strong risk factor for falls (2, 3), disability (4), low health-related quality of life, and premature death among older adults (5, 6). Skeletal muscle mass declines at an annual rate of 1-2% among adults over 50 years, leading to decreased muscle strength and function (7). The direct and indirect medical costs associated with sarcopenia are expected to rise rapidly in the era of population aging, resulting in a substantial financial burden for older adults, their caregivers, and the healthcare system. However, the molecular mechanism and etiology of sarcopenia remain unclear. Understanding the pathogenesis of sarcopenia is urgently needed to improve its prevention, diagnosis, treatment, and prognosis.

Metabolites represent the downstream expression of the genome, transcriptome, and proteome and are closely related to cellular phenotypes. Mass spectrometry-based metabolomics emerges as a robust approach to systematically analyze metabolic profiles in tissues, biological fluids, and cells (8), revealing the significance of metabolic flux in various diseases, including cancer (9), aging, and longevity (10, 11). Of the metabolites, the investigation of amino acids is essential. Understanding the amino acid profile changes may contribute to a better understanding of pathophysiological mechanisms.

Identifying amino acid profiles associated with sarcopenia may help identify at-risk populations, optimize prevention strategies, and develop new treatments. Previous studies have shown that changes in the plasma amino acid profile were associated with low muscle mass among older adults (12). The metabolism of amino acids, such as aspartic acid and glutamic acid, might play an essential role in regulating muscle mass and strength (13). There is evidence that lower blood levels of essential amino acids (EAA), branched-chain amino acids (BCAAs), especially leucine, were associated with lower skeletal muscle index (SMI), strength, and longer time to complete the chair stand (14). However, a study showed that the levels of isoleucine, leucine, tryptophan, serotonin, and methionine in the participants with low muscle quality were significantly higher than that in the participants with high muscle quality, which may be attributed to impaired metabolism of amino acids, resulting in reduced uptake of skeletal muscle, and thus increased circulating plasma amino acid levels (15). Inconsistencies in amino acid profiles in patients with sarcopenia will lead to variations in clinical practice and research.

Therefore, we conducted a systematic review and meta-analysis to evaluate and synthesize the evidence regarding the association between amino acid profile and sarcopenia in the present study.

## Methods

### Data Sources and Search Strategy

A comprehensive search was conducted on MEDLINE, EMBASE, and Cochrane library *via* Ovid SP for all publications related to amino acid metabolomics biomarkers of sarcopenia reported from inception to June 1, 2021. The terms sarcopenia, metabonomics, metabolomics, amino acid profile, and mass spectrometry were searched alone or in combination. Take the MEDLINE search policy as an example. The search strategies are presented in **Table 1**. A manual search for additional potentially applicable studies was carried out by using references of included studies. No language limitations were applied. The study followed the Preferred Reporting Items for Systematic Reviews and Meta-Analyses (PRISMA) flow diagram and checklist (16).

**Table 1:** Search strategy.

MEDLINE(R) Database: Ovid MEDLINE(R) Epub Ahead of Print, In-Process & Other Non-Indexed Citations, Ovid MEDLINE(R) Daily, and Ovid MEDLINE(R)	
1	exp Sarcopenia
2	(sarcopeni* or myopeni* or dynapeni*).ti,ab.
3	[(muscle or muscular) adj2 (atroph* or wasting* or weak or loss*)].ti,ab.
4	exp Metabolomics
5	exp Metabonomics
6	(metabolo* OR metabolomic* OR metabonomic* OR liquid chromatogra* OR gas chromatogra* OR ultra-performance liquid chromatograph* OR high performance liquid chromatograph* OR high-performance liquid chromatograph*).ti,ab.
7	(metabolit OR lipidomic OR UPLC OR proton nuclear magnetic resonance OR proton NMR OR nuclear magnetic resonance spectrometry OR H NMR OR mass spectrometry OR nuclear magnetic spectroscopy OR metabolic profiling OR amino acid profile OR amino acid metabolomics OR amino acid metabonomics OR amino acid metabolism).ti,ab.
8	(1 or 2 or 3) and (4 or 5 or 6 or 7)

Asterisks (*) indicate truncation symbol.

### Inclusion and Exclusion Criteria

We used the following inclusion criteria for each included manuscript: (1) Studies must have been conducted on adults (age ≥ 50 years). (2) A definition of sarcopenia was described in the methods section. The diagnostic criteria for sarcopenia included muscle mass, muscle strength, and/or body function. (3) It was an original article, including observational studies (cross-sectional studies, cohort studies, and case-control studies) and experimental studies (randomized controlled trials).

Articles that met the following criteria were excluded: (1) Studies performed in animals, case reports, reviews, conference abstracts, letters to the Editor, and books. (2) Therapeutic trials and articles used methods other than metabolomics.

### Data Extraction and Quality Assessment

Two investigators (MD and TL) independently filtered these articles’ titles and abstracts and retrieved the full text based on our inclusion and exclusion criteria to ensure accuracy. Disagreement in screening the articles was resolved by discussion between the two investigators, and if necessary, involving a third investigator (JY).

Two investigators extracted and summarized study characteristics in a standard form independently. Any disagreement was resolved by consensus. The following information was extracted from the eligible studies: name of the first author, year of publication, study population, study design, sample size, the mean age of individuals, percentage of females, diagnostic methods and specific criteria for sarcopenia, assessment method of muscle strength and muscle mass and physical performance, metabolomics techniques and metabolite targets, biological specimen (plasma or serum), and major metabolite outcomes. The quality of cross-sectional studies was assessed according to the Agency for Healthcare Research and Quality (AHRQ) checklist. There are 11 items in the AHRQ checklist; each item has three responses: “Yes,” “No,” and “Unclear”. A score of 0 was assigned to an answer of “No” or “Unclear”, while a score of 1 was given to an answer of “Yes”. Study quality was assessed based on the total score (range: 0-11) and classified into three categories: low (score: 0-3), moderate (score: 4-7), and high (score: 8-11).

### Data Synthesis and Analysis

We extracted the mean and standard deviation of concentrations of amino acids from each study. The median (range) was converted into average and standard deviation following a method published previously (17). We used the random effects models, which considered between- and within-study variability, to pool the association estimates between concentrations of amino acids and sarcopenia. We used the Cochran Q test (P <0.1 indicates statistically significant heterogeneity) and the I^2^ statistic (I^2^ >50% indicates statistically significant heterogeneity) to evaluate study heterogeneity. Forest plots were used to visualize the results. Standardized mean difference (SMD) with 95% confidence interval (CI) was selected for the continuous outcomes. As a sensitivity analysis, we restricted the analyses to studies in which muscle mass was measured by bioelectrical impedance analysis (BIA). We only analyzed amino acid metabolites that were used in at least two studies. We did not assess publication bias because fewer than 10 eligible studies were included. All statistical analyses were performed using Stata, version 12. A two-tailed P value of <0.05 was considered statistically significant.

## Results

### Literature Information

**Figure 1:**
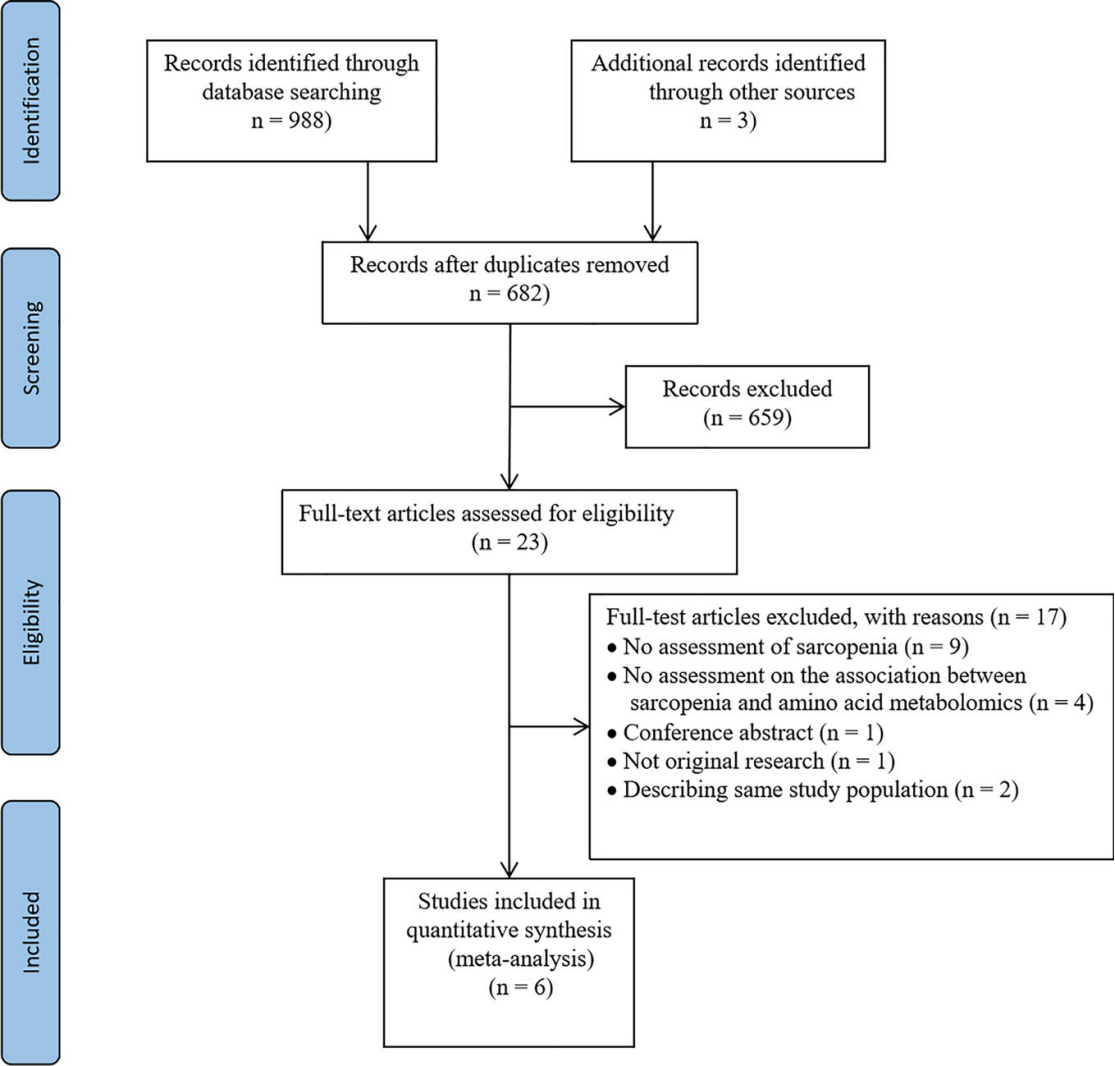
Flow of the search strategy and included studies.

In the initial search, 991 articles were retrieved from MEDLINE, EMBASE, and Cochrane. Six hundred and eighty-two studies were identified after eliminating duplicates. We further excluded 659 studies after following the review of titles and abstracts because they were not relevant to the subject, or were conducted in animals, or were non-original studies. Twenty-three articles were selected for careful full-text screening. Full-text articles were further excluded if they were not relevant to the topic or did not meet inclusion criteria. In total, six full-text articles were included in this systematic review and meta-analysis (**Figure 1**).

### Characteristics of Included Studies

Of the 991 screened papers, six papers were eligible (**Figure 1**). A total of 1,120 participants were included. All studies compared the amino acids levels in those with sarcopenia (case subjects) *versus* those without (control subjects). Sample size ranged from 28 to 90 in the case groups (n=358) and 30 to 327 in the control groups (n=762). Five studies included participants of both sexes; one study included women only (18). Five studies used LMM in combination with low physical performance (LPP) and/or low muscle strength (LMS) as the criteria for diagnosing of sarcopenia (18–22), while one study used only LMM as the diagnostic criterion (23). Muscle mass was measured by BIA in three studies (18, 19, 21), dual-energy X-ray absorptiometry (DXA) in two studies (20, 22), and computed tomography in one study (23). Muscle strength was measured through handgrip dynamometry in three studies (18, 19, 21), and lower limb strength in one study (20). Physical performance was measured *via* gait speed in five studies (18–22). Muscle mass, muscle strength, and physical performance were assessed according to cut-off thresholds recommended by the Asian Working group for Sarcopenia in three studies (18–20), European Working Group for Sarcopenia in Older people (EWGSOP) in one study (21), and the Foundation for the National Institutes of Health (FNIH) in one study (22), while only one study evaluated sarcopenia by measuring psoas muscle area at the caudal end of the third lumbar vertebra by computed tomography (CT) without fulfilling specific guidelines (23). Metabolite analysis was performed using blood samples; five used plasma and one used serum. All studies used targeted metabolomics for analyzing metabolite features. One study used high-performance liquid chromatography-tandem mass spectrometry (HPLC-MS/MS) (19), one study employed nuclear magnetic resonance spectroscopy (NMR) (21), and one study used ultra-performance liquid chromatography-mass spectrometry (UPLC-MS) (22), while the techniques used in other studies were unclear. Five studies’ main results were presented as mean and standard deviation (18, 20–23), and the remaining one study reported the correlation between sarcopenia and metabolites with median (range) (19). The characteristics of each eligible study are summarized in **Table 2**.

**Table 2:** Description of the studies included in the meta-analysis.

Reference	Population	Study design	Mean Age (years)	Sample Size	Female (%)	Diagnostic criteria for sarcopenia	Muscular strength	Lean Mass	Physical Performance Assessment	Metabolomics technique	Sample	Outcomes (Metabolites associations)
Yamada M et al. 2018 (18)	Japan	Cross sectional	Case 83.1±6.2 Control 76.0±6.5	Case 49 Control 94	Case 100 Control 100	AWGS criteria	Handgrip dynamometry	BIA	5m walking test	Unclear, targeted metabolomics	Plasma	(↓) Leucine, BCAAs, and EAAs
Toyoshima K et al. 2017 (19)	Japan	Cross sectional	Case 85.1±4.8 Control 76.1±6.3	Case 28 Control 132	Case 64.3 Control 65.2	AWGS criteria	Handgrip dynamometry	BIA	6m walking test	HPLC-ESI-MS, targeted metabolomics	Plasma	(↑) Glutamine, proline (↓) histidine, tryptophan
Lu Y et al. 2019 (20)	Singapore	Cross sectional	Case 73.9±5.3 Control 72.5±5.3	Case 87 Control 102	Case 63.2 Control 62.7	AWGS criteria	lower limb strength	DXA	6m walking test	Unclear, targeted metabolomics	Plasma	(↓) leucine, isoleucine, valine, Lysine, methionine, threonine, and phenylalanine
Ottestad I et al. 2018 (21)	Norway	Cross sectional	Case 78 (74-82) Control 74 (71-77)	Case 90 Control 327	Case 76.7 Control 45.6	EWGSOP criteria	Handgrip dynamometry	BIA	4m walking test	NMR spectroscopy, targeted metabolomics	Plasma	(↓) Leucine, isoleucine, valine , and BCAAs
Calvani R et al. 2018 (22)	Italy	Cross-sectional	Case 76.4±4.9 Control 74.6±4.3	Case 38 Control 30	Case 65.8 Control 53.3	FNIH criteria	Unclear	DXA	SPPB	UPLC-MS, targeted metabolomics	Serum	(↑) Asparagine, glutamic acid(↓) methionine
Toshima T et al. 2015 (23)	Japan	Cross-sectional	Case 54.8 ±9.0 Control 55.0 ± 10.4	Case 66 Control 77	Case 31.8 Control 62.3	Unclear	Unclear	psoas muscle area by CT	Unclear	Unclear, targeted metabolomics	Plasma	(↓) Leucine, isoleucine, and glutamine

AWGS, Asian Working group for Sarcopenia; EWGSOP, European Working Group for Sarcopenia in Older people; FNIH, the Foundation for the National Institutes of Health; BIA, Bioelectrical impedance analysis; DXA, Dual X-ray absorptiometry; CT, computed tomography; SPPB, Short physical performance battery; UPLC-MS, ultra-performance liquid chromatography-mass spectrometry; HPLC-MS, high-performance liquid chromatography-electrospray ionization tandem mass spectrometry; NMR, nuclear magnetic resonance; BCAA, branched-chain amino acid; EAA, essential amino acid. Arrows (↑) indicate positive association and Arrows (↓) indicate inverse association.

### Quality Assessment

We assessed the quality of all six eligible studies according to the AHRG checklist. All studies were of moderate quality (score: 3-6); five studies had a score of 5 and one scored 6 (**Table 3**).

**Table 3:** AHRQ checklist for assessing the quality of cross-sectional studies.

Study	Q1	Q2	Q3	Q4	Q5	Q6	Q7	Q8	Q9	Q10	Q11	Score	Quality
(18)	+	+	+	U	U	+	−	+	−	+	−	5	Moderate
(19)	+	−	+	U	U	+	−	+	−	+	−	5	Moderate
(20)	+	−	+	U	U	+	−	+	−	+	−	5	Moderate
(21)	+	+	+	U	U	+	+	+	−	+	−	6	Moderate
(22)	+	+	+	U	U	+	−	−	−	+	−	5	Moderate
(23)	+	−	+	U	U	+	+	−	−	+	−	5	Moderate

AHRQ, Agency for Healthcare Research and Quality; +, yes; -, no; U, unclear.

### Meta-Analysis of Remarkable Metabolites

**Figure 2:**
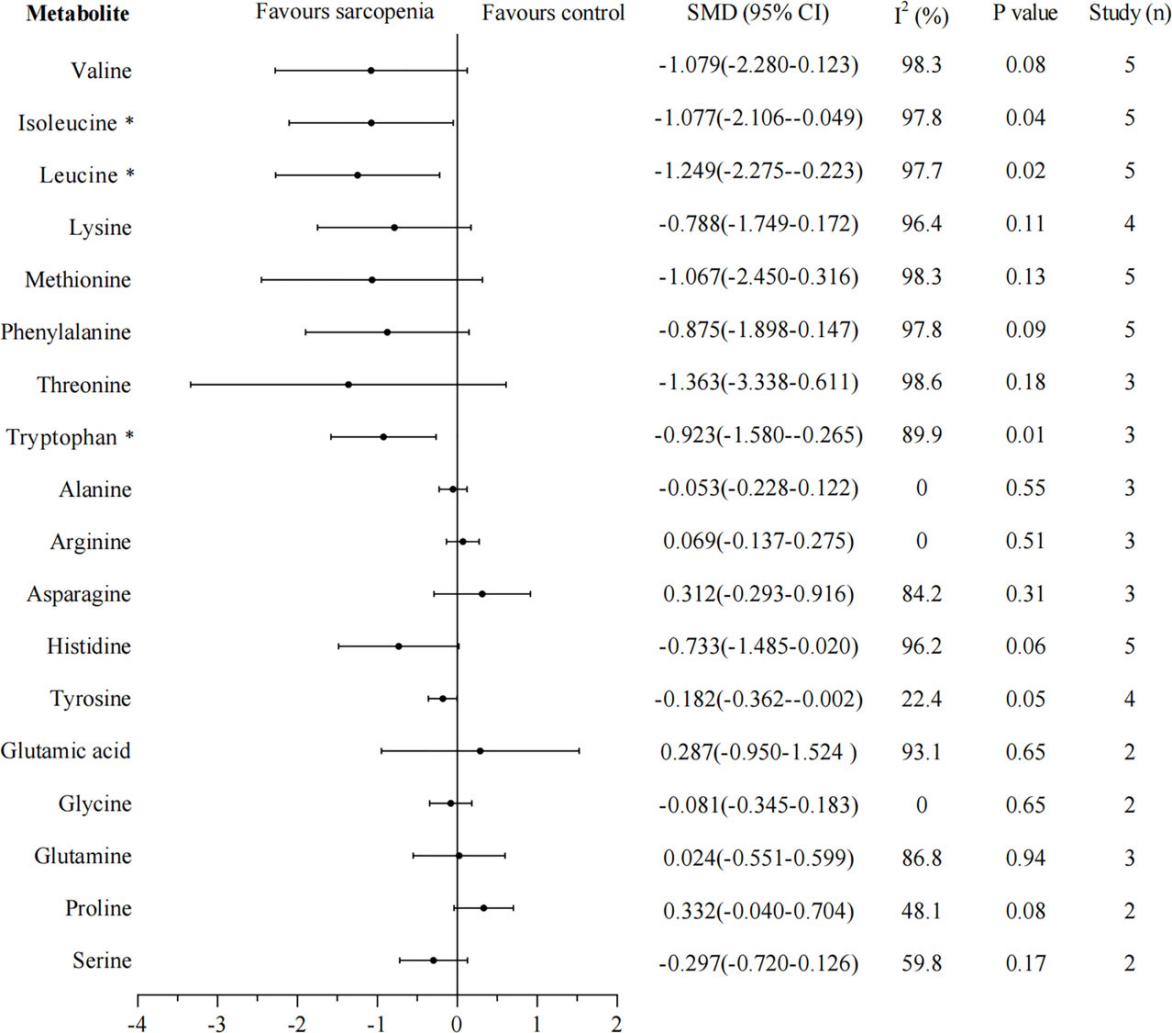
Forest plot for amino acid metabolomics in sarcopenic individuals *vs*. non-sarcopenic individuals. Overall estimates were obtained from forest plots of the meta-analysis using the random-effects model. Closed circles and horizontal bars represent the overall estimate and 95% CI. SMD, standardized mean difference; CI, confidence interval. Asterisks (*) indicate P-value <0.05.

Eighteen metabolites that were included in at least two studies were further analyzed by pooled meta-analysis using a random-effects model (**Figure 2**). The concentrations of BCAAs leucine (SMD -1.249, 95% CI: -2.275, -0.223), isoleucine (SMD -1.077, 95% CI: -2.106, -0.049), and aromatic amino acid tryptophan (SMD -0.923, 95% CI: -1.580, -0.265) were significantly lower in participants with sarcopenia than in those without, but the heterogeneity was high for each amino acid (leucine: I^2^ = 97.7%; isoleucine: I^2^ = 97.8%; tryptophan: I^2^ = 89.9%).

### Sensitivity Analysis

**Figure 3:**
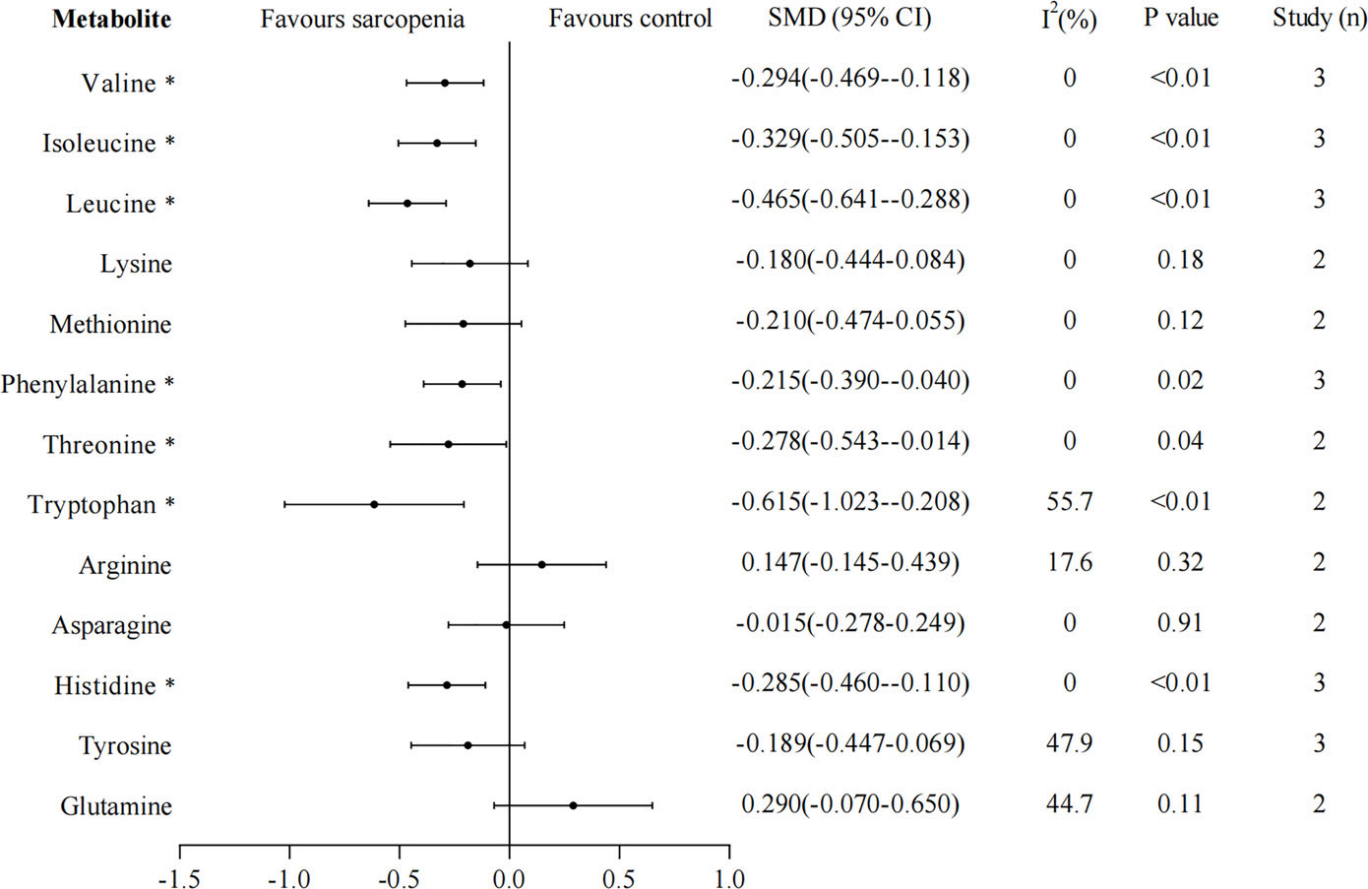
Sensitivity analyses for amino acid metabolomics in sarcopenic individuals *vs*. non-sarcopenic individuals (restricting to studies using bioelectrical impedance analysis to measure muscle mass). Overall estimate obtained from forest plots of the random-effects meta-analysis using the SMD. Closed circles and horizontal bars represent the overall estimate and 95% CI. SMD, standardized mean difference. CI, confidence interval. Asterisks (*) indicate P-value <0.05.

We rerun the random effects model excluding studies that did not measure muscle mass through BIA. Thirteen metabolites that were included in at least two studies were analyzed separately. The results of leucine, isoleucine, tryptophan, lysine, methionine, arginine, asparagine, tyrosine, and glutamine were unchanged, and the heterogeneity greatly reduced in studies except for arginine and tyrosine, I^2^ were 0%, 0%, 55.7%, 0%, 0%, 17.6%, 0%, 47.9%, 44.7%, respectively (**Figure 3**). However, the results of valine, phenylalanine, threonine, and histidine changed, their concentrations were significantly lower in sarcopenia than in non-sarcopenia (valine: SMD -0.294, 95% CI: -0.469, -0.118; phenylalanine: SMD -0.215, 95% CI: -0.390, -0.040; threonine: SMD -0.278, 95% CI: -0.543, -0.014; histidine: SMD -0.285, 95% CI: -0.460, -0.110), these four metabolites showed no significant evidence of heterogeneity (All I^2^ = 0%).

## Discussion

We performed a meta-analysis of literature related to sarcopenia’s metabolic profiling and highlighted the clinical significance of amino acids flux in sarcopenia. Our results from cross-sectional studies support inverse associations of the BCAAs leucine, isoleucine, and aromatic amino acid tryptophan with sarcopenia, which may provide insights into the development of sarcopenia and contribute to the prevention and treatment of sarcopenia.

### Compared With Other Studies

**Figure 4:**
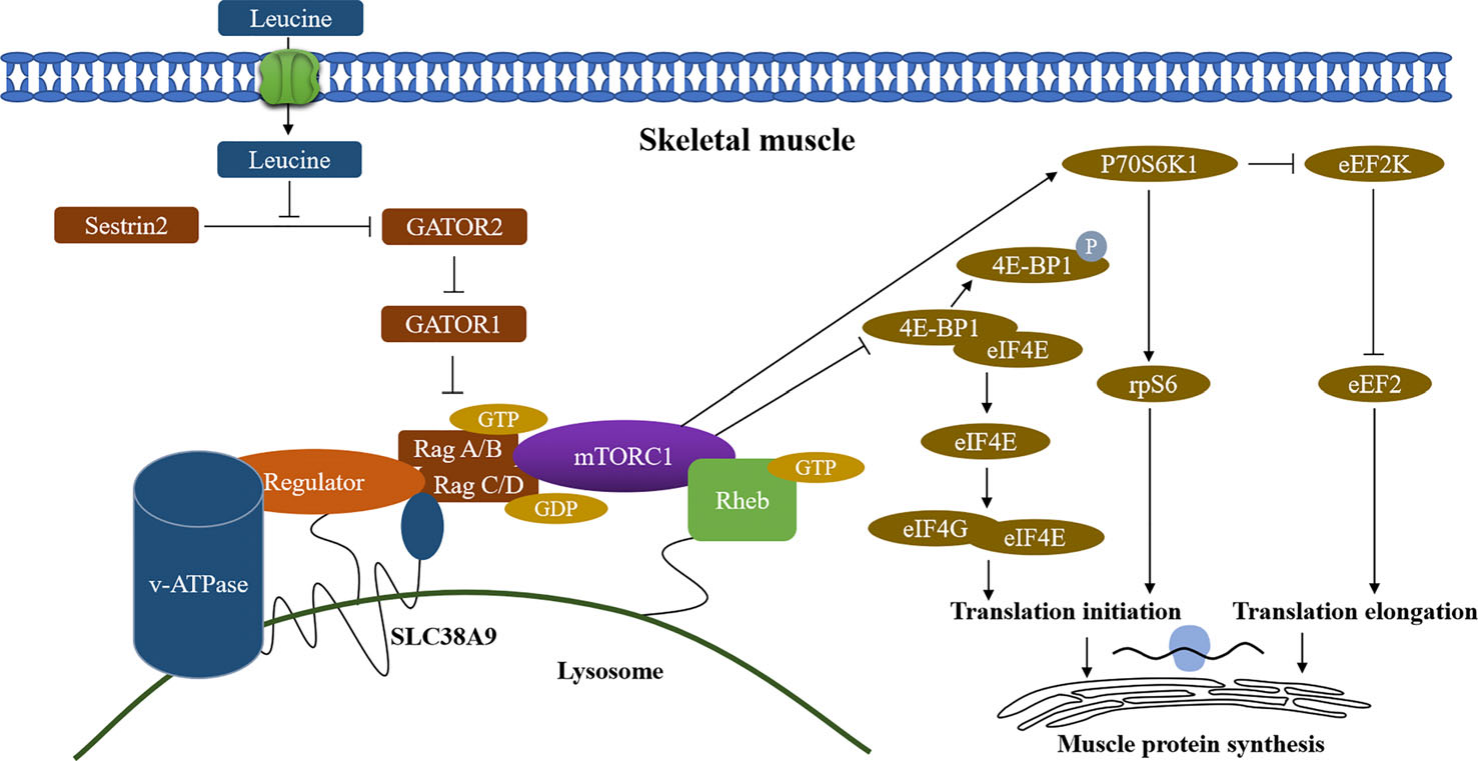
Regulation of skeletal muscle protein synthesis by leucine. Sestrin2, stress response protein 2; GATOR1/2, GAP activity toward Rags 1/2; mTORC1, mammalian/mechanistic target of rapamycin complex 1; v-ATPase, vacuolar H+ -adenosine triphosphatase; Rag A/B C/D, RAS-related GTP-binding protein A/B C/D; SLC38A9, Solute Carrier Family 38 Member 9; 4E-BP1, 4E-binding protein 1; eIF4E, eukaryotic initiation factor 4E; eIF4G, eukaryotic initiation factor 4G; p70S6K1, p70 ribosomal protein S6 kinase 1; eEF2K, eukaryotic elongation factor 2 kinase; rpS6, ribosomal protein S6; eEF2, eukaryotic elongation factor 2; Arrows (↑) indicate stimulation and blocked lines (⊣) indicate inhibition.

The significant roles of amino acids in maintaining skeletal muscle function and muscle protein synthesis have been disclosed in previous studies (24–26). In functionally limited older adults, the BCAA levels were significantly positively associated with thigh muscle cross-sectional area (CSA) and fat-free mass index (FFMI) (12). In our study, we found that leucine and isoleucine levels but not valine in participants with sarcopenia were significantly lower than those with non-sarcopenia, but high heterogeneity occurred across the integrated literature. Leucine and isoleucine are important essential amino acids that can only be obtained from food and are important nutritional factors for humans and animals (27). Studies have shown that the target of rapamycin complex 1 (mTORC1) kinase is a sensor of amino acids. Sestrin 1-3 interact with GATOR2 to negatively regulate the amino acid-sensitive pathway upstream of mTORC1 (28, 29). Whereas leucine stimulation leads to the dissociation of Sestrin 2 from GATOR2 and allows the activation of mTORC1 pathway (30), which further activates of eukaryotic initiation factor (eIF) 4E binding protein-1 and ribosomal protein S6 kinase (S6K1), and promotes the muscle protein synthesis (**Figure 4**) (31–34). Additionally, Liu S et al. (35) revealed that isoleucine increased the protein level of important myoblast genes and promoted myoblast proliferation and myoblast differentiation, suggesting that it has a potential function in myogenesis. A study indicated that the reduced muscle protein synthesis in the old adults could be reversed by the intake of additional leucine and the leucine-rich mixture of EAAs (36). Furthermore, with physical exercise, leucine-rich EAA supplementation effectively improved skeletal muscle mass volume, muscle strength, and walking speed in sarcopenic women (37). These findings suggest that decreased leucine and isoleucine concentrations may contribute to sarcopenia development and maintaining optimizing concentrations of EAAs in plasma, especially leucine and isoleucine, may be beneficial to muscle protein synthesis and very important for maintaining physical function in the sarcopenic elderly.

The aromatic amino acids include tryptophan, phenylalanine, and tyrosine, of which tryptophan and phenylalanine are EAAs. A previous study has shown that tryptophan can significantly affect muscle mass by its metabolite serotonin, and animals deficient in tryptophan showed low growth hormone (GH) levels and significant muscle atrophy (38). Dukes A et al. (39) confirmed that tryptophan stimulates skeletal muscle IGF1/p70s6k/mTOR signaling *in vivo* and can induce the expression of myogenic factors (myogenin, myoD, and myosin heavy chain) in C2C12 myoblasts *in vitro*, which play a prominent role in the regulation of myofiber size and muscle mass. Serum tryptophan decreases with age in older men (40). In our study, plasma tryptophan concentration was significantly lower in sarcopenic people than in non-sarcopenic people. Also, there is evidence that dietary supplementation with tryptophan could stimulate muscle protein synthesis in swine (38). It may be possible that the decrease of tryptophan leads to increased skeletal muscle cell atrophy and affects muscle mass, thus promoting sarcopenia. Therefore, we infer that the tryptophan metabolic pathway may be a promising target for preventing or treating skeletal muscle atrophy. Nevertheless, the findings were presented in cross-sectional studies. Thus, a longitudinal study is needed to clarify the role of tryptophan in the development of sarcopenia.

### Heterogeneity and Sensitivity Analysis

The reasons for the high heterogeneity may be that: (1) Methods of measuring muscle mass were different. A meta-analysis showed that BIA yielded higher sarcopenia prevalence estimates than DXA (41). Psoas is a minor muscle, it does not represent the whole muscle, and the cut-off point for low muscle mass is not well defined for the measurement of psoas muscle area by CT (42). (2) The included studies were carried out in different places (such as communities, rural areas, hospitals, or nursing homes) and different countries, so ethnic and regional factors might also influence the results. (3) Different metabonomics profiling techniques were applied, and the metabolomics results may have testing errors. Compared with other techniques, NMR analysis requires a larger sample size, poor isolation, and background noise that may obscure potential biomarkers (43).

For the high heterogeneity, we conducted sensitivity analysis by excluding studies that did not use BIA to measure muscle mass. It showed that the results of leucine, isoleucine, tryptophan, lysine, methionine, arginine, asparagine, and glutamine were consistent with those of the preliminary analysis. However, the concentrations of valine, phenylalanine, threonine, and histidine in the case group became significantly lower than those in the control group. Sensitivity analysis also revealed that the heterogeneity greatly was reduced in studies, suggesting that these deleted studies may lead to high heterogeneity and instability of results. Whether valine, phenylalanine, threonine, histidine, and tyrosine are potential biomarkers for sarcopenia requires further studies. Due to the limited number of included articles, other causes of heterogeneity could not be analyzed.

### Strengths and Limitations

To our knowledge, this was the first systematic review and meta-analysis to explore the association between amino acids and sarcopenia. Two investigators conducted the literature search, article screening, and data extraction independently to minimize bias. However, this study is not without limitations. First, our study’s most significant limitation was that the metabolomics results showed that the high heterogeneity indexes accounted for 2/3. Second, our results are subjective to selection bias. The participants were mostly volunteers, not randomly selected, and there were more women than men in these studies. Third, although our search strategy is not limited to English, studies that were not indexed in EMBASE, MEDLINE, or Cochrane library might be excluded. Finally, all of these included studies were cross-sectional, and we were unable to identify the causal relationship between amino acid metabolites and sarcopenia.

### Conclusion

In conclusion, reduced plasma concentrations of leucine, isoleucine, and tryptophan may signify sarcopenia. Determination of the optimal levels of plasma leucine, isoleucine, and tryptophan helps prevent and treat sarcopenia and maintain muscle mass and function. These findings need to be confirmed by further studies, including randomized trials and cohort studies.

## Data Availability Statement

The original contributions presented in the study are included in the article/supplementary material. Further inquiries can be directed to the corresponding authors.

## Author Contributions

MD designed the study protocol, extracted the data, designed and performed the analyses, interpreted the results, wrote the first drafts and revisions of manuscripts. TL contributed to the search strategy, study selection and extracted the data. JY revised drafts of the report, solved all disagreements and supervised the study. LD contributed in the interpretation of the findings and revised drafts of the report. All authors contributed to the article and approved the submitted version.

## Funding

This study was supported by grants from National Key R&D Program of China (No.2020YFC2005600 and 2020YFC2005602), and 1.3.5 project for disciplines of excellence, West China Hospital, Sichuan University (NO. ZY2017201).

## Conflict of Interest

The authors declare that the research was conducted in the absence of any commercial or financial relationships that could be construed as a potential conflict of interest.

## Publisher’s Note

All claims expressed in this article are solely those of the authors and do not necessarily represent those of their affiliated organizations, or those of the publisher, the editors and the reviewers. Any product that may be evaluated in this article, or claim that may be made by its manufacturer, is not guaranteed or endorsed by the publisher.
